# Treatment of Bites of Dogs

**Published:** 1912-12

**Authors:** 


### Treatment of Bites of Dogs.

The dog should not be killed, but chained up and kept under close observation for a period of sixteen days at the least. If he is infected with rabies, he will show symptoms in that time, and in all probability will die within ten days. It is a fatal disease in animals. Pasteur treatment, to be effective, must be instituted within five days after the infliction of the bite, or its usefulness is very much impaired. In doubtful cases, or where the animal has disappeared, it is better to give the treatment than to take chances, because the virus is harmless if given under aseptic precautions, and if the animal happens to have been rabid we have probably saved the patient from a dreadful death.—Tarbell, in *Jour. Med. Soc. New Jersey.*

